# Expanding the phenotype: Four new cases and hope for treatment in Bachmann‐Bupp syndrome

**DOI:** 10.1002/ajmg.a.62473

**Published:** 2021-09-03

**Authors:** Elizabeth A. VanSickle, Julianne Michael, André S. Bachmann, Surender Rajasekaran, Jeremy W. Prokop, Ruben Kuzniecky, Floris C. Hofstede, Katharina Steindl, Anita Rauch, Mark H. Lipson, Caleb P. Bupp

**Affiliations:** ^1^ Division of Medical Genetics and Genomics Spectrum Health and Helen DeVos Children's Hospital Grand Rapids Michigan USA; ^2^ International Center for Polyamine Disorders Grand Rapids Michigan USA; ^3^ Department of Pediatrics and Human Development, College of Human Medicine Michigan State University Grand Rapids Michigan USA; ^4^ Department of Neurology–Northwell Health Zucker Hofstra School of Medicine New York New York USA; ^5^ Department of General Pediatrics, Wilhelmina Children's Hospital University Medical Centre Utrecht Utrecht The Netherlands; ^6^ Department of General Pediatrics, Wilhelmina Children's Hospital Institute of Medical Genetics, University of Zurich Zurich Switzerland; ^7^ Genetics Department Kaiser Permanente Sacramento California USA

**Keywords:** alopecia, Bachmann‐Bupp syndrome, DFMO, macrocephaly, *ODC1*, polyamines

## Abstract

Bachmann‐Bupp syndrome (BABS) is a rare syndrome caused by gain‐of‐function variants in the C‐terminus of ornithine decarboxylase (ODC coded by the *ODC1* gene). BABS is characterized by developmental delay, macrocephaly, macrosomia, and an unusual pattern of non‐congenital alopecia. Recent diagnosis of four more BABS patients provides further characterization of the phenotype of this syndrome including late‐onset seizures in the oldest reported patient at 23 years of age, representing the first report for this phenotype in BABS. Neuroimaging abnormalities continue to be an inconsistent feature of the syndrome. This may be related to the yet unknown impact of ODC/polyamine dysregulation on the developing brain in this syndrome. Variants continue to cluster, providing support to a universal biochemical mechanism related to elevated ODC protein, enzyme activity, and abnormalities in polyamine levels. Recommendations for medical management can now be suggested as well as the potential for targeted molecular or metabolic testing when encountering this unique phenotype. The natural history of this syndrome will evolve with difluoromethylornithine (DFMO) therapy and raise new questions for further study and understanding.

## 1 INTRODUCTION

Ornithine decarboxylase 1 *(ODC1)* is a key gene coding for an enzyme (ODC) in the polyamine (PA) pathway, a metabolic system important for embryogenesis, organogenesis, and neoplastic cell growth (Casero Jr. & Marton, 2007; Gerner & Meyskens Jr., 2004; Pegg, 2016; Pegg & McCann, 1982; Wallace et al., 2003). A recently established OMIM entry (#619075) called Bachmann‐Bupp Syndrome (BABS) is caused by gain‐of‐function variants in the C‐terminus of ODC and is characterized by alopecia, developmental delay, macrosomia, macrocephaly, and some dysmorphic features (Bupp et al., 2018; Rodan et al., 2018). Laboratory analysis of patient samples demonstrated elevated ODC protein, ODC enzymatic activity levels, and abnormal PA levels (i.e., increase in putrescine) in affected patients. Treatment of patient‐derived primary dermal fibroblasts with ODC inhibitor eflornithine (aka difluoromethylornithine; DFMO) showed normalization of these levels (Schultz et al., 2019). This suggested the potential for treatment as DFMO has previously been shown to be tolerated in the treatment of African sleeping sickness (trypanosomiasis) (Alirol et al., 2013; Priotto et al., 2009) and pediatric neuroblastoma (Lewis et al., 2020; Saulnier Sholler et al., 2015). In addition, a previously developed transgenic mouse model showed alopecia with resolution after treatment with DFMO (Megosh et al., 1995; Soler et al., 1996). Adding genotype and phenotype information from four additional patients to the five individuals already reported helps further characterize this syndrome and its natural history, which is of particular importance in lieu of the potential for treatment early in the disease course.

## 2 CASE PRESENTATIONS

### 2.1 Patient 6

**FIGURE 1:**
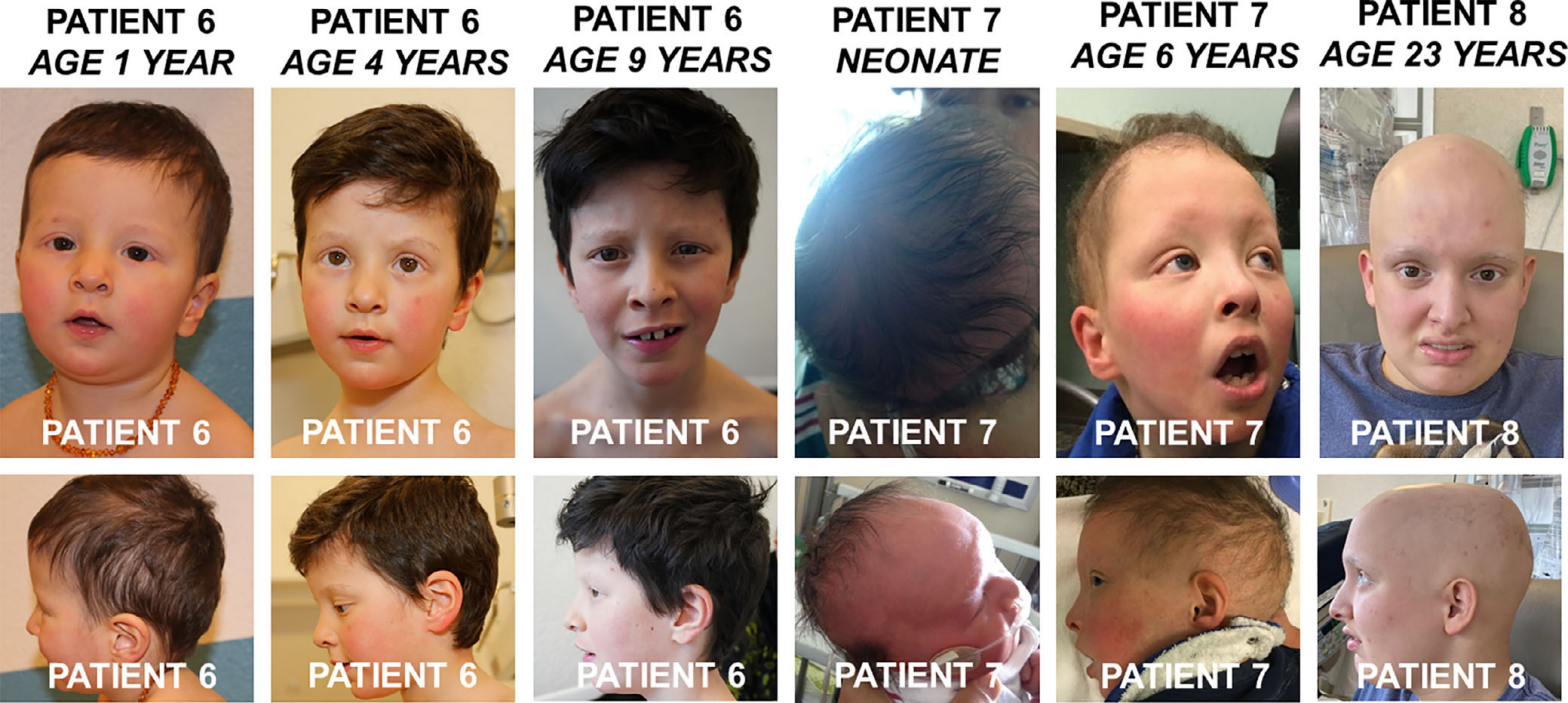
Representative clinical phenotypes from cases 6 to 8. The first three panels show the physical facial phenotypes of the patient presented in Case 6 at ages 1, 4, and 9 years. The fourth panels demonstrate the hair loss seen from birth (top panel) to age 1 month (bottom panel) for the patient presented in Case 7, as well as the physical facial phenotype of the same patient in the fifth panels. The last panels show the physical facial phenotypes as well as hair loss of the patient presented in Case 8 at age 23 years

This patient is a 9‐year‐old Caucasian boy at the time of reporting and was born at 39 weeks gestation after an uncomplicated pregnancy. At birth, he measured at the 87th percentile (52 cm) for height, 36th percentile (3170 g) for weight, and greater than 36th percentile (34 cm) for head circumference based on World Health Organization (WHO) 0–2 years standard growth curves. Examination at age 9 identified height at the 42nd percentile (134 cm), weight in the 23rd percentile (26,400 g), and head circumference at the 90th percentile (54.4 cm) based on CDC 2–18 years standard growth curves. He walked independently at 25 months, spoke his first words at 3 years, and was toilet trained at 9 years. At 4.5 years, he could speak in 3–4‐word sentences, exhibiting limited verbal capacity. He is reported to have an intellectual disability with an IQ of 55 and behavioral concerns that include stubbornness, although he has good social skills. Brain MRI identified hypoplastic chiasma, tractus opticus, and a small pituitary gland. Past medical history also includes hypotonia, ataxia, significant constipation, keratosis pilaris on the upper and lower limbs and cheeks, significantly reduced sweating, and reduced sensitivity to pain. Dysmorphic facial features include sparse eyebrows and eyelashes, sparse scalp hair only in early childhood with current scalp hair being shaggy and dry, broad forehead, elongated face, low anterior hairline, infraorbital fold, hypertelorism, narrow bridge of nose, thick and anteverted nasal alae, low insertion of the columella, deep and short philtrum, everted lower and tented upper lip vermillion, prominent maxillary central secondary incisor teeth and diastema of the upper and lower central incisors, prominent and pointed chin, and large earlobes (Figure 1). Other distinctive physical characteristics are hypertrichosis on the back, pectus carinatum superior with excavatum inferior, winged scapulae, bilateral cubitus valgus, long fingers and broad terminal phalanges, and bilateral genu varum.

He has a history of a previous nondiagnostic chromosomal microarray. He was identified to have a de novo heterozygous variant in the *ODC1* gene, c.1242‐2A>G, (IVS11‐2A>G) that is predicted to destroy a canonical splice acceptor site in intron 11. This variant is not observed in large population cohorts (Lek et al., 2016; 1000 Genomes Consortium, 2015; Exome Variant Server).

### 2.2 Patient 7

This patient is a 6‐year‐old Caucasian boy at the time of reporting and was born after a pregnancy complicated by polyhydramnios. He was born at 38 weeks with APGAR scores of 3 and 7. At age 5, his height was in the 29th percentile, weight at the 11th percentile, and head circumference at the 42nd percentile. He spoke his first words at 4.5 years, was not walking yet at examination, with a degree of intellectual disability. Brain MRI at age 2 identified non‐specific T2 hyperintensities, but was otherwise unremarkable. Small atrial communication was identified on echocardiogram at age 4. Past medical history also includes hypotonia, left gaze prominence, nutrition drink and protein shake supplementation due to poor feeding and failure to thrive, constipation, gastroesophageal reflux, ventricular septal defect that spontaneously closed, and syringohydromyelia that resolved by age 3. Hair at birth was notable for band of alopecia between sparse hair on calvaria and neck which fell out within the first month of life (Figure 1). Sparse hair is now present throughout the scalp with very little eyebrow hair and eyelashes. He has a history of recurrent follicular cysts on the head, ears, and axilla, some of which have required surgical drainage. Dysmorphic features include a long face, broad forehead, sagittal craniosynostosis, cryptorchidism, and choanal atresia.

Prior whole‐exome sequencing (WES) was non‐diagnostic. Whole‐genome sequencing (WGS) through the Undiagnosed Diseases Network demonstrated a de novo heterozygous variant in the *ODC1* gene, c.1313_1316delCTGT (p.438Rfs*9), which causes a disruption in this reading frame, leading to the entire DNA sequence following the mutation to be read incorrectly. This variant is absent in large population cohorts (Broad gnomAD database).

### 2.3 Patient 8

This patient is a 23‐year‐old Caucasian male at the time of reporting and was born after a pregnancy complicated by cephalopelvic disproportion. He was born at 40 weeks with APGAR scores of 8 and 9. At birth, he was reported to be in the 99th percentile (55 cm) for height and 95th percentile (4218 g) for weight based on WHO 0–2 years standard growth curves. He was noted to be macrocephalic (>99.9% WHO) at birth (40 cm), which has been a consistent finding on his physical exam since. He is reported to have lack of motor coordination, independent walking at 4 years old, as well as oro‐motor dyspraxia and speaking his first words at age 6. He is reported to have moderate intellectual disability, being unable to engage in linear conversation. At age 19, he had a psychological evaluation that diagnosed him with anxiety, disruptive behavior, mild aggression, poor social and coping skills, difficulties in academic performance, and self‐injurious behaviors. A series of brain MRIs have consistently identified nonspecific white matter signal changes as well as parenchymal and hippocampal volume loss advanced for age. This patient was diagnosed with absent seizures at age 14 years. At that time, he was placed on sodium valproate and remained seizure free for 2 years, but the treatment led to fatty liver and hair loss. No controlling medication has since been identified, with vagal nerve stimulator implanted at age 20, which exacerbated the seizures and recent ketogenic diet that showed no improvement. He has multiple seizure types but atypical absence, atonic, and generalized tonic–clonic are common. Most recent EEG performed at age 23 identified severely abnormal EEG with multiple short bursts of low voltage generalized spikes, abundant multifocal spikes, and generalized background slowing. This patient's history is significant for alopecia affecting the scalp, eyelashes, eyebrows, and axillary and pubic hair with sparse arm and leg hair that was initially reversible but has been stably at a loss in recent years. Previous medical history includes hypotonia and skin findings including a hemangioma, pigmented nevus, and multiple scattered hyperpigmented macules. Dysmorphic facial features noted include bilateral epicanthal folds, high palate, slight facial asymmetry with drooping of the left mouth corner, frontal bossing, dolichocephaly, and kyphosis (Figure 1).

High hCG and low uE3 on prenatal triple screen caused the family to pursue a prenatal karyotype, which was normal. At age 11, *PTEN* testing was negative. At age 10, microarray identified a 12p13.32 duplication, which was later identified to also be present in his asymptomatic father. This finding was replicated on microarray ordered at age 19. Also, at age 19, he had normal very long chain and branched‐chain fatty acid analysis as well as normal free and total carnitine. WES at age 20 identified a homoplasmic *MT‐ATP8* m.8553C>T variant of uncertain significance, *RTTN* c.2738T>C paternal variant of uncertain significance*, RTTN* c.5572C>G maternal variant of uncertain significance, *RTTN* c.590G>A maternal variant of uncertain significance, and heterozygous de novo variant in the *ODC1* gene, c.1242‐2A>G (IVS11‐2A>G). The *ODC1* variant is predicted to destroy a canonical splice acceptor site in intron 11. This variant is not observed in large population cohorts (Lek et al., 2016; 1000 Genomes Consortium, 2015; Exome Variant Server).

### 2.4 Patient 9

This patient is a 1.5‐year‐old Caucasian female at the time of reporting and was born at 41 weeks gestation after an uncomplicated pregnancy with APGAR scores of 6, 6, and 6. At birth, she required resuscitation with oxygen, bag‐mask ventilation, and eventual CPAP. She was admitted to the NICU for a possible perinatal infection. Her birth weight was in the 87th percentile (3780 g) and head circumference was greater than 99th percentile (38.5 cm) using WHO 0–2 years standard growth curves. Height measured on day 14 of life was in the 99th percentile (54 cm) using WHO 0–2 years standard growth curves. Examination at 10 months identified height in the 98th percentile (75.3 cm), weight in the 73rd percentile (9500 g), and head circumference greater than the 99th percentile (49 cm) using WHO 0–2 years standard growth curves. She was noted to have axial hypotonia which delayed her walking until 17 months. At the time of reporting, she does not have any words. Brain MRI identified frontal cortex decreased gyration, germinal cysts, and left porencephalic cyst, and frontal and parietal white matter hyperintensity. Echocardiogram identified mild pulmonary stenosis with mild insufficiency. Dysmorphic features include sparse hair and eyebrows that are improving with time, hypertelorism, mild retrognathism, broad forehead, and macrocephaly.

Prior genetic testing included a microarray, which identified an 83 kb duplication of 18q21.32. Trio WES demonstrated a de novo heterozygous variant in the *ODC1* gene, c.1252C>T (p.Gln418*), which causes premature termination of the protein. This variant is not observed in large population cohorts (Broad gnomAD database).

## 3 RESULTS

Adding these new patients to those five individuals previously reported (Bupp et al., 2018; Rodan et al., 2018), patients with BABS now total 6 males and 3 females. All variants are de novo with no patients having a second contributory genetic diagnosis. Previous genetic testing is variable with most patients having nondiagnostic chromosomal microarray. Metabolic testing was performed on some, and 2 of 4 newly reported patients had previous WES that was nondiagnostic with a diagnosis made for one patient on re‐analysis after initial description of this syndrome and the other by WGS. Polyhydramnios was seen in the majority of patients (four of seven) and gestational age appears to be within normal limits. Head circumference appears to be larger at birth, along with length but not typically weight. The one patient without macrocephaly has sagittal craniosynostosis, which has not been surgically corrected. Postnatal complications are not consistent. Global developmental delay is uniform with this syndrome, typically in the more severe range with associated behavior concerns. The earliest walking was at 17 months but often not until 3–4 years of age. Speech appears limited with first words at 2–3 years and some patients remain nonverbal. Muscular hypotonia is also a common feature in all eight living individuals. Every patient had a brain MRI performed at some time point, and neuroimaging abnormalities are common, but not with a particular pattern or recurrence of findings.

Alopecia is a notable feature of this condition, particularly since this is not present at birth, but often occurs shortly after birth with hair loss in large sections. (Figure 1, Patient 7). Description of physical features does not suggest a particular pattern of dysmorphism of all recognizable facial features except for the broad forehead/macrocephaly. Cryptorchidism is present in three of six males. Cardiac, pulmonary, or hepatic involvement seems to be variably present. Constipation, a common ailment and even more common in general with patients with developmental delay, is present in three of seven patients (Table 1).

**TABLE 1:** Patient genotypes and phenotypes

	Patient 6	Patient 7	Patient 8	Patient 9	Bupp et al. (2018)	Rodan et al. (2018)–1	Rodan et al. (2018)–2	Rodan et al. (2018)–3	Rodan et al. (2018)–4
Variant NM_001287190.1	c.1242‐2A>G (IVS11‐2A>G)	c.1313_1316delCTGT (p.Pro438Argfs*9)	c.1242‐2A>G (IVS11‐2A>G)	c.1252C>T (p.Gln418*)	c.1342A>G (p.Lys448*)	c.1241+1G>T (IVS11+1G>T)	c.1240_1241dupTG (p.Trp414Cysfs*17)	c.1255C>T (p.Gln419*)	c.1242_1263del22 (p.Trp414*)
Inheritance	De novo	De novo	De novo	De novo	De novo	De novo	De novo	De novo	De novo
Age at most recent evaluation	9 years 5 months	5 years 9 months	20 years	10 months	32 months	6 years	16 years	8 years	34 weeks gestational age—stillborn fetus
Sex	Male	Male	Male	Female	Female	Male	Female	Male	Male
Prenatal findings	None	Polyhydramnios	Cephalopelvic disproportion	Normal	Polyhydramnios, decreased fetal movement	Polyhydramnios, decreased fetal movement	Polyhydramnios	Normal	Polyhydramnios, large for gestational age, macrocephaly, neuroimaging abnormalities
Head circumference	90% (54.4 cm)	42%	99%	99% (49 cm)	90%–97%	93%	>97%	Mean	99%
Height	42% (134 cm)	29%	77% (182 cm)	98% (75.3 cm)	50%–75%	29%	50%–75%	Mean	>97%
Weight	23% (26,400 g)	11%	88% (85,700 g)	73% (9500 g)	10%–50%	32%	25%–50%	Mean	97%
Global developmental delay	Yes	Yes	Yes	Yes	Yes	Yes	Yes	Yes	N/A
Age at walking	25 months	Not yet	4 years	17 months	Not yet	3 years	3.5 years	2 years	N/A
Age at first words	3 years	4.5 years	6 years	Not yet	Not yet	29 months	N/A (nonverbal)	3 years	N/A
Behavior	Stubborn	—	Anxiety, disruptive behavior, mild aggression, poor social and coping skills, poor academic performance, self injurious	None	Autism	ADHD, aggression	ADHD, aggression	No concerns	N/A
Epilepsy	No	No	Yes—onset at 14 years	No	No	No	No	No	N/A
Hypotonia	Yes	Yes	Yes	Yes	Yes	Yes	Yes	Yes	N/A
Dysmorphic features	Broad forehead, elongated face, low anterior hairline, hypertelorism, narrow bridge of nose, thick and anteverted nasal alae, deep and short philtrum, everted lower and tented upper lip vermillion, prominent and pointed chin, large earlobe, pectus carinatum superior and excavatum inferior, winged scapulae, bilateral cubitus valgus, long fingers and broad terminal phalanges, bilateral genu vara	Broad forehead, craniosynostosis, elongated face, cryptorchidism, choanal atresia	Frontal bossing, dolichocephaly, bilateral epicanthal folds, high palate, slight facial asymmetry (drooping of left mouth corner), kyphosis	Broad forehead, hypertelorism, mild retrognathia	Cupped ears, high arched palate	High forehead, hypertelorism, ptosis, deep‐set eyes, 5th finger clinodactyly, fusion of first and seconds ribs, cryptorchidism	High and broad forehead, hypertelorism, deep‐set eyes, blepharophimosis, tapering of the arms and legs	High forehead, hypertelorism, mild ptosis, down‐slanting palpebral fissures, cryptorchidism	Hypertelorism, thin upper lip
Skin	Keratosis pilaris upper and lower limbs and face	Recurrent follicular cysts on head, ears and axilla, erythematous follicular pattern on face	Irregular border in the interscapular region, 2 mm pigmented nevus on right occiput, 1‐8 mm hyperpigmented macules over abdomen/ back/inner left thigh		Recurrent follicular cysts on neck and back, erythematous vascular marking on posterior head and neck, subcutaneous vasculature on scalp became more prominent over time	Dry skin, keratosis pilaris, ulerythema ophryogenes	Normal	Normal	N/A
Hair	Sparse scalp hair in early childhood (15mo), currently shaggy and dry scalp hair, hypertrichosis on the back, sparse eyebrows and eyelashes	Born with dark hair on calvaria and lower neck with band of alopecia between which fell out during first month of life, sparse scalp hair now regrown, sparse eyebrows and eyelashes	Alopecia affecting scalp, eyelashes, eyebrows, axillary and pubic hair as well as sparse arm and leg hair	Sparse hair and eyebrows	Born with copious silver/blonde‐colored hair which fell out during the first month of life, no eyebrows and few eyelashes congenitally	Absent eyebrows, sparse eyelashes	Absent eyebrows, sparse eyelashes, sparse scalp hair	Absent eyebrows, sparse eyelashes	Sparse/absent eyebrows and eyelashes
Other	Intellectual disability, severe constipation, significantly reduced sweating, reduced sensitivity to pain	Intellectual disability, small atrial communication, spontaneously resolved VSD, resolved syringohydromyelia, left gaze prominence, poor feeding, constipation, GERD, failure to thrive, brittle fingernails	Moderate intellectual disability, megalencephaly, prominent ventricles (R>L)	~83 kb microduplication of 18q21.32, mild pulmonary stenosis with mild insufficiency, mild constipation as neonate with easy vomiting	DHCR7 carrier, intellectual disability, spasticity, intermittent esotropia, pseudostrabismus, bilateral myopic astigmatism, right‐sided sensorineural hearing loss, G‐tube placement for feeding support, exaggerated curve to nails and brittle when cut, reduced sensitivity to pain	ESES on EEG, myringotomy, celiac disease, hypoplastic toenails	Myringotomy, constipation	Myringotomy, hypoplastic nails	None
MRI, brain	Hypoplastic chiasma and tractus opticus, small pituitary	Non‐specific T2 hyperintensities	Overall brain parenchymal volumes reduced for age, mild bilateral hippocampal volume loss, non‐specific white matter signal changes	Decreased gyration frontal cortex, germinal cysts and left porencephalic cyst, frontal and parietal white matter hyperintensities	Prominent cystic changes in periventricular region, resolution of previous periventricular cystic changes but progression of white matter volume loss in both cerebral hemispheres, enlarged lateral ventricles	Bilateral germinolytic cysts, peritrigonal white matter volume loss, dysmorphic rostrum and genus of corpus callosum, mildly dysmorphic cerebellar vermis	Thickened foreshortened corpus callosum, numerous dilated perivascular spaces, mild prominence of lateral ventricles	Dilated perivascular Spaces, diffuse white matter signal abnormality	Dilated perivascular spaces, ventriculomegaly, white matter abnormalities, periventricular cysts, focal polymicrogyria, intracranial calcification

**FIGURE 2:**
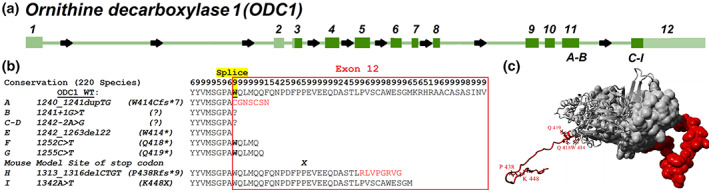
*ODC1* gain‐of‐function variants for all nine cases thus far. (a) Exon map of the *ODC1* gene, where exon 3–12 code for the ODC protein (dark green). Light green represents the untranslated regions of the gene. (b) Sequence alignment of ODC and resulting patient proteins. Conservation is shown on the top for 220 vertebrate species on a scale of 1–9 with 9 highest. Splice site of exons 11 and 12 is shown in yellow. The position of the gain‐of‐function mouse model stop codon is marked with X. (c) Protein model of the ODC dimer with exon 12 sequence marked in red

Novel findings in these newly reported patients include the first report of seizures, with onset at age 14 years. These seizures have been resistant to treatment. This patient presented as Patient 8, 23 years old at reporting, is also the oldest patient now known to have this syndrome. Genotype findings continue to show variants in the C‐terminus of the ODC protein. In the biochemical pathway, destabilization of this region blocks protein degradation and leads to ODC accumulation. This results in elevation in putrescine and acetyl‐putrescine, but not other PAs. Finally, we also observe the first recurrent variant for this syndrome (c.1242‐2A>G; p.IVS11‐2A>G) in Patients 6 and 7 (Figure 2).

## 4 DISCUSSION

The additional patients described here along with the oldest reported patient, allow for a broader description of the spectrum of this disorder and expansion of the history. Common findings include developmental delay, hypotonia, and varying degrees of alopecia, typically soon after birth. All patients underwent brain imaging with the larger head size and developmental delay being the indication. The findings on imaging however appear to be inconsistent. Brain development is a complex process, and the ubiquitous functionality of the PA pathway lends itself as a potential target for downstream abnormality. Further understanding of the mechanism for brain anomalies in this syndrome is necessary, particularly considering that it could potentially impact treatment. Patient 8 is notable for developing epilepsy, but the later onset could mean that the younger patients are at risk for future development of seizures. Anti‐epileptic and Vagus Nerve Stimulation (VNS) treatment has had a limited impact with the seizures being resistant to ketogenic diet. The initiation of this diet is challenging at best and the variable PA content of various foods may present a unique conundrum for clinicians in the future (Muñoz‐Esparza et al., 2019). It remains unclear whether therapies that are directed at PA levels have a role to play in controlling seizures.

Macrocephaly continues to be a consistent finding in most cases; however, macrosomia now does not. At this time, it would be a reasonable to consider macrocephaly to be a key feature of the syndrome while viewing macrosomia with less certainty. Further correlation of birth growth parameters and serial monitoring of height, weight, and head circumference may inform this over time. The finding of craniosynostosis in one patient without macrocephaly does explain lack of macrocephaly, although craniosynostosis has not been seen in other patients. The role of PAs in growth may relate to patient size at birth and throughout life, potentially impacted as well by developmental delay and the impact of that on feeding and nutrition.

All diagnoses to date have been made using broad sequencing, such as WES or WGS. The potential for this syndrome to be suggested by metabolic testing is limited by the availability of PA‐specific testing assays. Global metabolomic testing could raise the possibility of this diagnosis. As both molecular and metabolomic testing capabilities expand in scope and access, as well as costs for both potentially decrease, more individuals of BABS may be identified. The natural history of rapid hair loss shortly after birth appears to be unique to this syndrome and could be a reason for consideration of targeted *ODC1* gene analysis when observed in any patient with developmental delay.

Recommendations for screening and management for BABS can start to be formulated. Much of this is more generalizable to other neurodevelopmental disorders, such as the risk of vision and hearing concerns requiring screening or the potential for constipation possibly related to the common finding of hypotonia. No consistent concerns were noted on echocardiogram or abdominal imaging, suggesting the need only for symptomatic management. Developmental assessment and intervention, such as physical and speech therapy are recommended; particularly as developmental milestones vary in patients. Additional support may be required for more severely affected patients. As more is known about treatment with DFMO, the natural history of neurocognitive findings in this syndrome may change (Rajasekaran et al., 2021). Diagnoses like autism spectrum disorder (ASD) and intellectual disability (ID) may become more apparent as cognitive and behavioral development progresses functionality to the level of being able to make that diagnosis with more confidence.

## 5 CONCLUSION

Four new patients of BABS further confirm the phenotypic description of a recognizable syndrome characterized by developmental delay, macrocephaly, and rapid appearance of alopecia shortly after birth. Causative variants cluster in the C‐terminus of the ODC protein. With further disease description and knowledge, more diagnoses may be made early in disease course allowing potential intervention with DFMO.

## CONFLICT OF INTEREST

André S. Bachmann, Surender Rajasekaran, and Caleb P. Bupp are listed inventors of a pending U.S. patent application based on the method of treating or preventing developmental disorders associated with mutations in the *ODC1* gene. All other authors declare no conflict of interest as it relates to the content in this article.

## AUTHOR CONTRIBUTIONS

Caleb P. Bupp designed the study. Floris C. Hofstede, Ruben Kuzniecky, Mark H. Lipson, and Katharina Steindl investigated the patients. Caleb P. Bupp, Julianne Michael, and Elizabeth A. VanSickle generated the first draft. Jeremy W. Prokop generated computational images of Figure 2. All authors reviewed and edited all drafts of the manuscript including the final draft.

## Data Availability

The data that support the findings of this study are available from the corresponding author upon reasonable request.
